# Design of a Super Twisting Sliding Mode Controller for an MR Damper-Based Semi-Active Prosthetic Knee

**DOI:** 10.3389/frobt.2022.870018

**Published:** 2022-05-19

**Authors:** Dawit Assfaw, Solomon Seid

**Affiliations:** ^1^ Faculty of Mechanical and Industrial Engineering, Bahir Dar Institute of Technology, Bahir Dar, Ethiopia; ^2^ Center for Research and Development, College of Engineering, Defense University, Bishoftu, Ethiopia

**Keywords:** magnetorheological damper, super twisting, sliding mode control, swing phase, semi-active

## Abstract

A large number of transfemoral amputees living in low-income countries could not access a much-needed prosthesis. Hence, affordable semi-active prosthetic knees have been designed in recent years. As the swing phase of the gait cycle is unstable as compared to the stance phase, these designs could not perfectly mimic this phase of a healthy human being. In contribution toward such a gap, this study proposes the modeling and design of a robust controller for magnetorheological (MR) damper-based semi-active prosthetic knee. A dynamic model representation for the swing phase of the single-axis knee is derived first. Subsequently, an MR damper valve model is developed. Then, a higher-order sliding mode controller is designed and evaluated for its stability and performance. The numerical simulation results show that the super twisting sliding mode controller improves the semi-active prosthetic knee’s tracking efficiency. The design exhibited the finest performance, providing a low normalized mean square error as compared to previous designs. The variable speed performance and robustness evaluation for this controller also showed its ability to continue providing excellent performance in the presence of disturbances.

## 1 Introduction

Transfemoral amputation is among the types of leg amputation which occur between the hip and knee joints. A recent study by the World Health Organization (WHO) states that 35–40 million amputees are living in developing countries, with knee amputation being the most common one. The most familiar causes of physical disabilities include traffic accidents, natural disasters, war, and the like. Those people with leg amputation will have reduced ability to move. Hence, a prosthetic knee is required to substitute the amputee limb to enhance the quality of their life by assisting them in performing activities such as standing and walking like wholesome people.

Throughout the years of prosthetic knee development, three types of knees have been studied to date, namely, active (powered), semi-active (adjustable damping), and mechanically operated passive prosthetic knees. The active prosthetic knee has its power supply to operate effectively. Amputees having this prosthetic knee will be able to perform complex activities such as walking up stairways and standing up. However, the main drawback of this prosthetic knee is its consumption of a large amount of energy and a large actuator, which leads to a heavy weight and reduction of battery life (Ekkachai and Nilkhamhang, 2016).

In contrast, mechanically passive knees do not need a power supply to function. They only provide a constant amount of damping suitable for only one speed of walking. Semi-active knees have several advantages over mechanically operated passive prosthetics, including improved knee stability, the capacity to change the walking pace, and the ability to react to changing environmental conditions (Ekkachai and Nilkhamhang, 2016).

Semi-active knees are mostly known for their use of magnetorheological (MR) fluid (MRF)-based mechanisms, for instance, MR dampers. As mechanically passive prosthetic knees cannot change damping values, they expose amputees to health problems and injuries using MRF-based dampers on prosthetic limbs, which deliver an extensive dynamic range and will avoid these conditions from occurring. Since an MR damper has a lot of advantages such as a small volume, a simple structure, low power consumption, and large resistance, it is considered to be a good alternative for the semi-active control of prosthetic limbs (Xie et al., 2015).

The prosthetic knees incorporating readily made MR dampers designed and manufactured by Lord Corporation have some drawbacks. Hence, the main purpose of the author (Seid et al., 2019b) was to propose an integrated design to address the drawbacks of these MRD-based prosthetic knees. This study has developed the overall design of prosthetic knees controlled by MRD. The swing-phase dynamic system modeled the knee and MR damper so that the control parameters are optimized using the constrained nonlinear optimization technique; then, the swing-phase trajectory produced by the MRD-based knee with optimized control parameters was compared with a healthy human swing-phase trajectory from experimental data. However, the work was not microcontroller-based and variable walking speed characteristics of the knee were not studied.

In another research, (Zuo et al., 2021), a second-order sliding mode controller was designed to control both the swing and stance periods of a transfemoral prosthetic knee. In the research, an MRD was designed and used as a controlling and damping force-providing mechanism. The work considered a flat ground walking condition at a 4.68 kmph walking speed. The design was tested in real-life applications using a prototype. The results show that the knee angle trajectory has a relatively high error of 9°. Even if the result is as close as the desired knee angle, the work did not consider the performance of the controller at various speeds, and the robustness was not checked for different walking conditions and walking speeds.

Another promising work (Ekkachai et al., 2014) proposed a new control algorithm for the swing phase of the variable damping prosthetic knee. This work applied neural network predictive control for the prosthetic knee, and the algorithm was tested at various walking speeds. It comprises a feedforward neural network (FNN) structured swing-phase model and predictive control which is optimized by a constrained nonlinear optimization technique. The constructed NNPC attempts to solve the setback of the MR damper, which was capable of only supplying a damping force, whereas the normal gait cycle needs active and damping forces simultaneously. The NNPC is capable of estimating the expected knee angle trajectory with a specified amount of command voltage input. However, it could not be applied in a real-world application (online) since it requires too much computation time.

The previous study by the author (Seid et al., 2016) considered the design and evaluations of two types of swing phase controllers, namely, an MRF-based damper and hydraulic damper, for the single-axis knee to control its swing phase. The assessment outcomes indicate a better capability of the MR damper over that of the hydraulic one. However, this research did not consider utilizing a microcontroller, and the controlling parameters of the damper were not checked for its performance while walking on a relatively rough terrain, jumping, and climbing and at variable walking speeds. Furthermore, the dynamic model of the single-axis knee proposed in this study did not consider the effect of the thigh angle while checking the performance of the designed MR damper. Hence, a modified model has to be developed to fully describe the relationship between the thigh and shank angle.

To solve the drawbacks which are found in most of the semi-active prosthetic knees, the authors (Seid et al., 2017; Seid et al., 2019b) have previously proposed a design model of an MR actuator for the prosthetic knee application as a swing-phase controller for amputees on a ground level walk. The proposed design shows its efficiency in closely mimicking the swing-phase trajectory of a healthy human being; however, for the amputee to walk on various terrains and walking scenarios, the designed damper should provide variable damping at various speeds since different terrains and walking speeds require a variable damping force to avoid the exposure of the amputees to walking discomfort leading to injuries and health problems, that is, due to undamped forces on the hip joint. Hence, a robust controller is required.

### 1.1 Article Structure

A modified dynamic model for the single-axis knee is developed by using Lagrangian mechanics in Section 2. Then, the resulting MRF is analyzed using the Bingham Plastic model accordingly in Section 3. Following this, Section 4 presents the design of super twisting SMC and its simulation on MATLAB. In Section 5, simulation results and comparisons are given and analyzed to verify the designed controller’s efficiency. Section 5 provides a discussion on the simulation results presented in Section 5. Finally, Section 7 concludes the article.

## 2 Modeling a Single-Axis Knee

**FIGURE 1:**
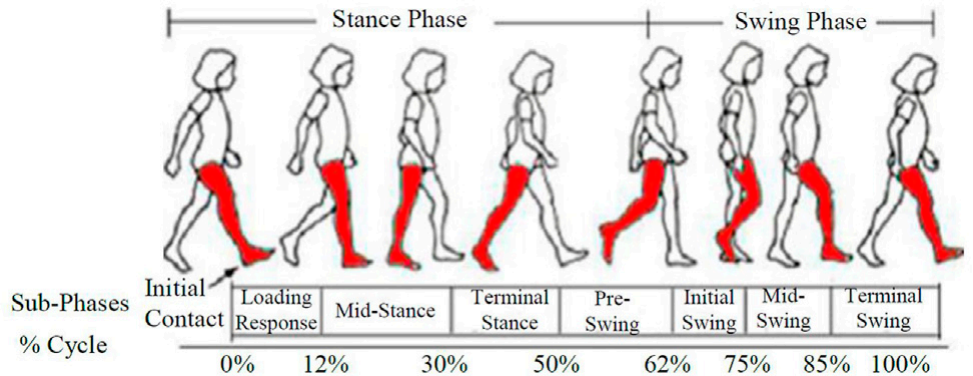
Gait cycle (Nandy et al., 2012).

During a regular gait cycle, cyclic occurrences can be seen when walking on a level terrain. Toe-off and foot strike are the two different occurrences that can distinguish the periodic pattern. One gait cycle is classified into two main phases/periods: stance and swing (Figure 1, right leg shaded). Stance is the time between when the foot first meets the ground and when it rises from it, and it accounts for 62% of the gait cycle. The rest 38% of the gait cycle is made up of the swing period when the foot leaves the ground and swings in the air (Nandy et al., 2012).

**FIGURE 2:**
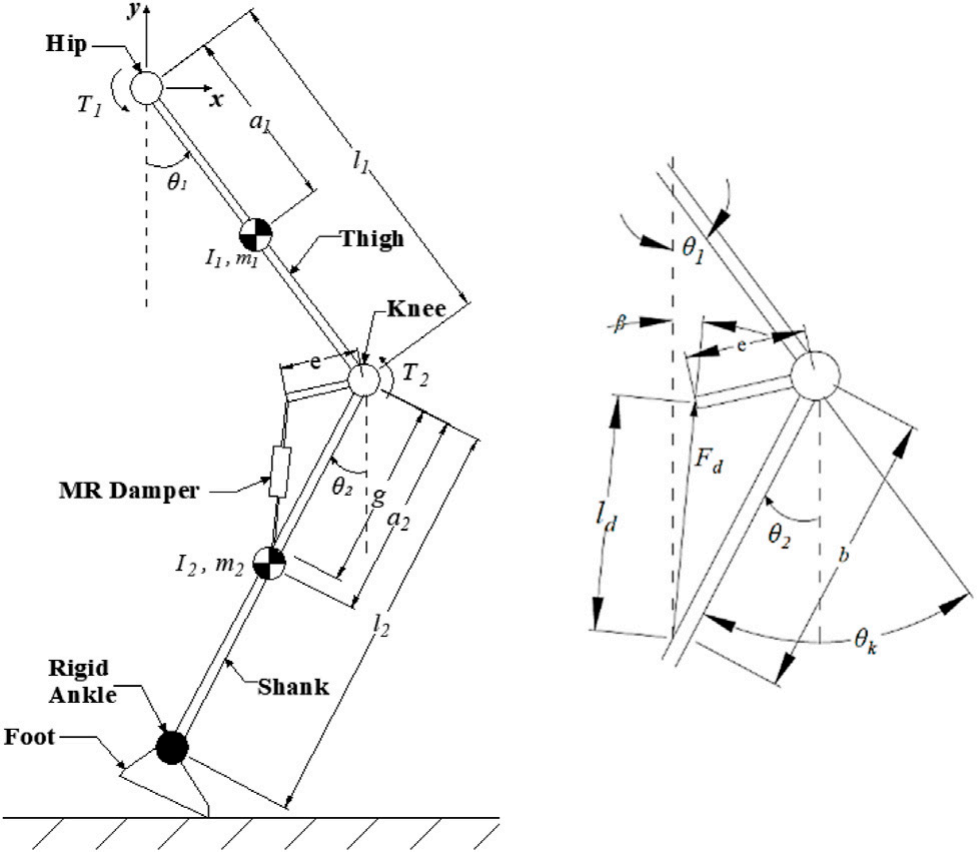
Swing-phase model and damper force and angle resolution.

The dynamic model of the swing-phase leg model with the prosthetic knee, depicted in Figure 2, is developed based on the following assumptions:

• The ankle is considered rigid and fixed on the shank, and the shank and thigh are assumed to be connected by pin joints.
• The thigh is allowed to have vertical and horizontal movement in two dimensions.
• The swing motion of the two-link rigid body chain is taken as a two-degree-of-freedom double pendulum.

The resulting swing-phase representation of the knee by applying Lagrangian formulation will be

$$T=M\left(\theta \right)\ddot{\theta }+V\left(\theta ,\dot{\theta }\right)+G\left(\theta \right).$$ (1)

From this relation, the inertia, Coriolis and centrifugal, and gravitational matrices are as follows:

$$\begin{array}{l} M\left(\theta \right)=\left(\begin{array}{cc} \left(m_{1}a_{1}^{2}+I_{1}+m_{2}l_{1}^{2}\right) & -\left(l_{1}a_{2}m_{2}C_{12}\right)\\ -l_{1}a_{2}m_{2}C_{12} & \left(m_{2}a_{2}^{2}+I_{2}\right) \end{array}\right),\\ V\left(\theta ,\dot{\theta }\right)=\left(\begin{array}{l} l_{1}a_{2}m_{2}S_{12}{\dot{\theta }}_{2}^{2}\\ l_{1}a_{2}m_{2}S_{12}{\dot{\theta }}_{1}^{2} \end{array}\right),\\ G\left(\theta \right)=\left(\begin{array}{l} \left(a_{1}m_{1}+l_{1}m_{2}\right)\left(gS_{1}+{\ddot{x}}_{H}C_{1}+{\ddot{y}}_{H}S_{1}\right)\\ m_{2}a_{2}\left(gS_{2}-{\ddot{x}}_{H}C_{2}+{\ddot{y}}_{H}S_{2}\right) \end{array}\right), \end{array}$$ (2)

where $m_{1}$ , *I*_*1*_, $m_{2}$ , and *I*_*2*_ are the masses and moments of inertia of the thigh and shank, respectively. $S_{1}$ , $C_{1}$ , $S_{2}$ , and $C_{2}$ are the sines and cosines of the thigh and shank angles, respectively. $S_{12}$ and $C_{12}$ are, respectively, the sine and cosine of summation of the thigh and shank angles. *a*_*1*_ and *a*_*2*_ are the distances from hip and knee joints to the center of masses of the high and shank, respectively. *g* is the gravitational acceleration. ${\ddot{x}}_{H}$ and ${\ddot{y}}_{H}$ are the hip horizontal and vertical accelerations, respectively. $\theta_{1}$ and $\theta_{2}$ are the thigh and shank angles, respectively; on the other hand, ${\dot{\theta }}_{1}$ and ${\ddot{\theta }}_{1}$ are the angular velocity and acceleration of the thigh, respectively; ${\dot{\theta }}_{2}$ and ${\ddot{\theta }}_{2}$ are the angular velocity and acceleration of the shank, respectively. *l*_*d*_ is the length of the damper.

From the damper resolution, the angle the damping force makes with the vertical axis is derived from the aforementioned figure to be

$$\beta =\left(\frac{1-\left(\frac{b}{e}\right)\sin\theta_{k}}{\sqrt{1+\left(\frac{b}{e}\right)-2\left(\frac{b}{e}\right)\sin\theta_{k}}}\right)-\theta_{k}-90,$$ (3)

where *e* is the extension length of the damper from the knee joint, *b* is the distance where the base of the damper is attached on the shank from the knee joint, and $\theta_{k}$ is the knee angle relative to the thigh axis.

## 3 Modeling the MR Damper

MRFs are members of the family of fluids whose properties are governed by the strength of the magnetic field. When subjected to a magnetic field, MRFs can convert from a linear viscous liquid to semi-solids, with the yield strength being controlled quickly and constantly. When a magnetic field is supplied to the suspended particles, they become polarized and migrate, reducing the ensemble’s stored energy. As the employed magnetic field increases, the energy desired to yield those chain-like arrangements will increase, making the fluid rigid to flow (Guglielmino et al., 2008).

Modeling MRF involves dealing with the rheological behavior of these materials in pre-yield and post-yield regimes. Two models have been used popularly to represent MRF models.

### Bingham Plastic Model

A quasi-static Bingham plastic model supposes that the fluid exhibits a shear stress comparative to the shear rate in the post-yield region, expressed by the following equations:

$$\tau =\eta \dot{\gamma }+\tau_{y}\left(H\right)\mathrm{sgn}\left(\dot{\gamma }\right),\;\;\left|\tau \right|\geq \tau_{y},$$ (4)

$$\tau =G\gamma ,\;\;\;\;\;\;\;\;\left|\tau \right|<\tau_{y},$$ (5)

where τ is the shear stress induced in the fluid, $\tau_{y}$ is the yield shear stress dependent on the magnetic field applied, *H*, η is post-yield viscosity (plastic viscosity) which does not depend on the magnetic field employed, $\dot{\gamma }$ represents the shear strain rate, *G* represents the complex shear modulus, and *sgn(* ^*.*^ *)* is the signum function (Seid et al., 2018). Eq. 4 governs the performance of the fluid in the magnetic field readily present, and Eq. 5 describes the viscoelastic behavior of the fluid (Jolly et al., 1999).

### Herschel–Bulkley Model

The Bingham plastic model does not consider the change of the viscosity of the fluid when the applied magnetic field varies; hence, another model was developed. The variation of viscosity in the post-yield region is clearly described by a more accurate Herschel–Bulkley model. When the MRF undergoes an increased shear rate, this model predicts post-yield shear thinning or thickening (Guglielmino et al., 2008; Seid et al., 2019b). The Herschel–Bulkley model is represented as follows:

τ=(K|γ˙|1m+τy(H)sgn(γ˙))sgn(γ˙), (6)

where *K* is the consistency index, *m* is the MRF behavior index, and m>1 in the aforementioned equation represents the shear-thinning fluid, whereas for m<1 , the equation will be used for shear-thickening fluids. The Bingham model is a linear variant of the Herschel–Bulkley model, as may be seen, that is, when *m = 1*. Even if the Herschel–Bulkley model represents the behavior of the MRF accurately, the Bingham plastic model is mostly used since it is straightforward (Marinca et al., 2017).

**FIGURE 3:**
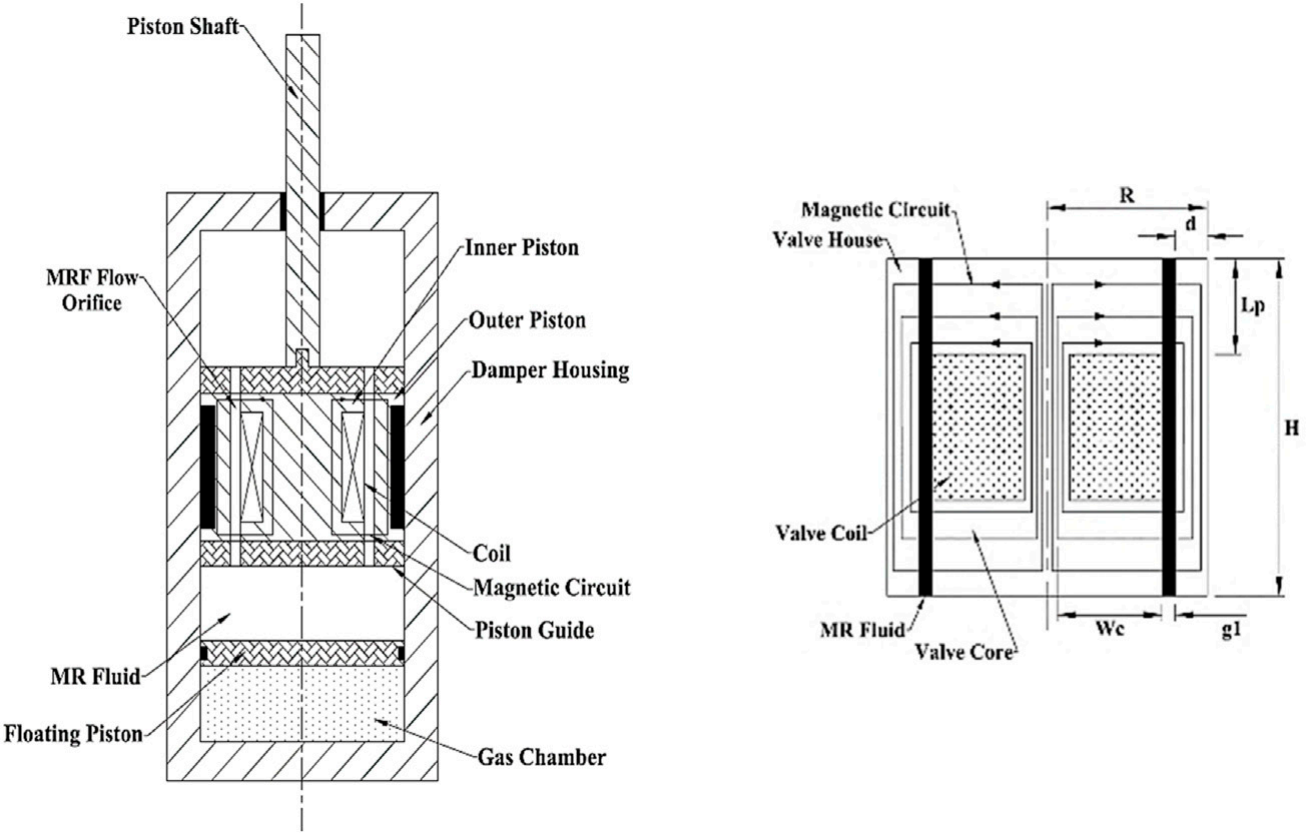
Diagram of MRD and the MR valve (Seid et al., 2019a).

Following the development of the MRF model, the modeling of the MR valve is described, supposing that the MRF displays Bingham plastic characteristics and that the flow in the valve paths is completely developed. The schematic diagram of the MR damper and its single-coil valve is shown in Figure 3.

Flow resistance which is dependent on the magnetic field is caused by magnetic field lines that are orthogonal toward the MRF’s direction of flow. A considerable amount of pressure drop is inevitable while the fluid passes through the valve, represented by Jolly et al. (1999)

$$\text{Δ}P=\text{Δ}P_{\eta }+\text{Δ}P_{\tau }=\frac{6\eta H}{\pi g^{3}R_{1}}Q+2c\frac{L_{p}}{g_{1}}\tau_{y}.$$ (7)

In Eq. 7, $\Delta P_{\eta }$ and the field-dependent yield stress component $\Delta P_{\tau }$ can be regulated by the strength of the directed magnetic field across the ducts of the valve. Q represents the volume flow rate, given by $Q=\left(A_{p}-A_{r}\right){\dot{x}}_{p}$ , η is the developed yield stress, and *R*_*1*_ is the duct’s average radius, which is the space between the centroid of the duct to the valve axis. Since, in this case, the duct is a rectangular annular duct, *R*_*1*_ *= R – d – 0.5g*_*1*_. *H*, *w*_*c*_, and *g*_*1*_ are the valve’s length, pole length, and orifice (fluid gap), respectively (Seid et al., 2019b). The parameter *c*, with a value between *2.07* and *3.07*, is a coefficient, represented by Seid et al. (2017).

$$c=2.07+\frac{12Q\eta }{12Q\eta +0.8\pi d^{2}\tau_{y}}.$$ (8)

The damping force produced by the damper is presented by using the following equation:

$$F_{d}=P_{a}A_{r}+\text{Δ}P\left(A_{p}-A_{r}\right).$$ (9)

Here, *A*_*p*_ and *A*_*r*_ are the piston and rod areas of the damper, respectively, and *P*_*1*_ and *P*_*2*_ represent the pressures in the lower and upper chambers, respectively. Now substituting Eq. 7into Eq 10,weget.

$$F_{d}=P_{a}A_{r}+\left(\frac{6\eta H}{\pi g^{3}R_{1}}\right){\left(A_{p}-A_{r}\right)}^{2}{\dot{x}}_{p}+2c\frac{L_{p}}{g_{1}}\left(A_{p}-A_{r}\right)\tau_{y}\cdot \mathrm{sgn}\left({\dot{x}}_{p}\right)$$ (10)

Finally, the most important parameter in controlling the damping force, yield stress (field-dependent) with respect to the employed field intensity, $\tau_{y}\left(H\right)$ , can be approximately expressed as follows:

$$\tau_{y}\left(H\right)=C_{o}+C_{1}H+C_{2}H^{2}+C_{3}H^{3},$$ (11)

where $C_{0},\;C_{1},\;C_{2},\;and\;C_{3}$ are curve-fitting parameters decided from an experiment using a curve-fitting algorithm. The MR damper valve has a single coil wrapped around it to create the desired amount of magnetic field using the input electric current. The coil is wound on the valve and connected to an electric source like a battery, and the valve material is then magnetized and behaves like a permanent magnet. To enhance the precision of the magnetic circuit solution, FEM is employed to analyze the precise distribution of field intensity along the active length where MRF is revealed to the magnetic field (Nguyen and Choi, 2009; Nguyen et al., 2009; Nguyen et al., 2014; Seid et al., 2018; Seid et al., 2019a). In this research, the magnetic analysis conducted to develop the relation between damping force and input current using commercial FEM software ANSYS by Seid et al. (2019a) is adopted.

From this optimal design of the MR damper valve, the curve-fitting relationship between the input current and yield stress can be derived to be

$$\tau_{y}=0.5275+5.32I-4.053I^{2}+1.018I^{3}\;\times 10\;kPa.$$ (12)

## 4 Super Twisting Sliding Mode Controller

Classified under the VSCS category, the sliding mode controller is by far one of the most robust controllers, with the characteristics of switching its control law that leads the system states to the anticipated sliding surface (Edwards, Alwi and Hamayun, 2018). Conventional sliding mode controllers operate by applying discontinuous high-frequency switching. However, high-frequency switching control needs to be reduced as much as possible, and continuous control is a better choice. Hence, super twisting SMC will be designed to overcome the drawbacks (Shtessel et al., 2014).

Now, the sliding variable can be defined using the following equation:

$$\sigma =\dot{e}\;+\;ce,\;\;c>0.$$ (13)

To derive the aforementioned sliding variable to zero in a finite amount of time, the following continuous control law can be applied:

$$u=c\sqrt{\left|\sigma \right|}\cdot sign\left(\sigma \right),\;\;c>0.$$ (14)

The compensated sliding variable dynamics will be

$$\dot{\sigma }=-c\sqrt{\left|\sigma \right|}\cdot sign\left(\sigma \right).$$ (15)

The specified control u forces the sliding variable to nil in a finite amount of time, *t*_*r*_. On the other hand, when $\varphi \left(y,\dot{y},t\right)\neq 0$ , the sliding variable dynamics becomes

$$\dot{\sigma }=\varphi \left(y,\dot{y},t\right)-c\sqrt{\left|\sigma \right|}\cdot sign\left(\sigma \right),$$ (16)

where σ and $\dot{\sigma }$ are the sliding variable and its derivative, respectively, $\varphi \left(y,\dot{y},t\right)$ is the cumulative disturbance term, *e* and $\dot{e}$ are the error and its derivative, respectively, and *u* is the control input.

However, due to the presence of a disturbance term, the sliding variable dynamics will fail to converge to zero using the disturbance-freederived control law of the form in Eq. 14; hence, it is desirable to add a term (*w*) in Eq. 14 so that it follows the disturbance term, $\varphi \left(y,\dot{y},t\right)\neq 0$ , in a finite amount of time, and the disturbance will then be reimbursed. After the disturbance has been removed, σ —dynamics will match with Eq. 15 and σ→0 in a finite amount of time. Now by taking $\left|\varphi \left(y,\dot{y},t\right)\right|\leq C$ and considering the aforementioned modification, the modified control law will be

$$\left\{ \begin{array}{l} u=c\sqrt{\left|\sigma \right|}\cdot sign\left(\sigma \right)+w,\\ \dot{w}=b\cdot sign\left(\sigma \right), \end{array}\right.\;c=1.5\sqrt{C};\;b=1.1\times C,$$ (17)

In this study, the error dynamics will be described by the following equation:

$$e=\theta_{k\left(desired\right)}-\theta_{k}.$$ (18)

## 5 Results

**FIGURE 4:**
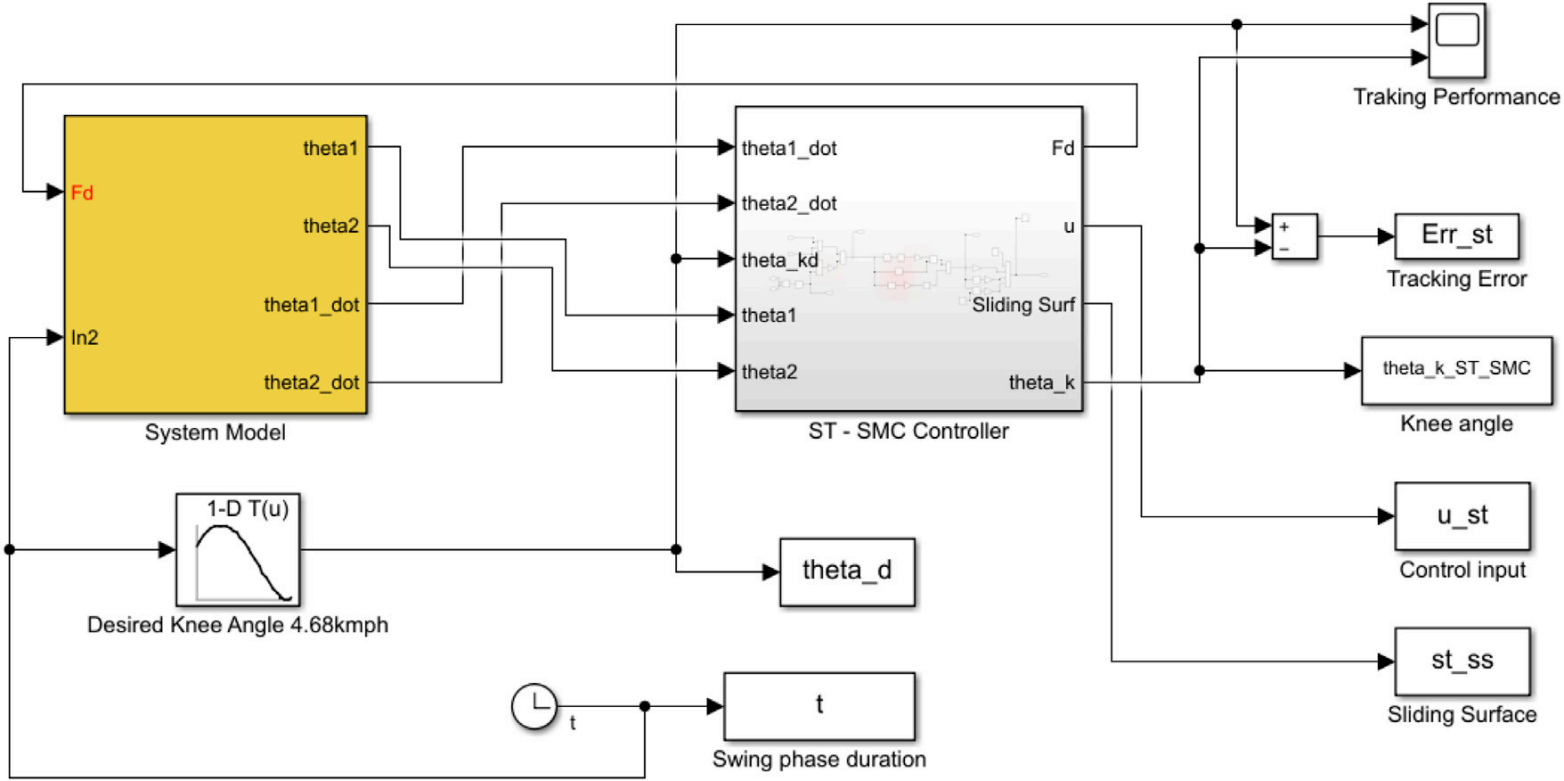
SIMULINK block diagram of the system model and ST-SMC controller.

The simulation of the designed controllers proceeds in place. The schematic block diagram used in the simulation of the controller’s performance is provided in Figure 4 below.

The physical parameters of an average human being with a mass of 56.7 kg are listed in Table 1.

**TABLE 1:** Physical parameter of a healthy human being (Seid et al., 2016).

Symbol	Value	Symbol	Value
I_1_	0.058 kg m^2^	a_1_	0.136 m
I_2_	0.108 kg m^2^	a_2_	0.2576 m
m_1_	5.67 kg	l_1_	0.314 m
m_2_	3.46 kg	l_2_	0.425 m

**FIGURE 5:**
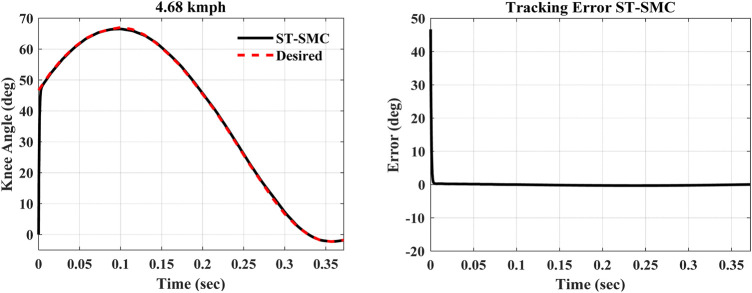
Tracking performance and error of the controller.

**FIGURE 6:**
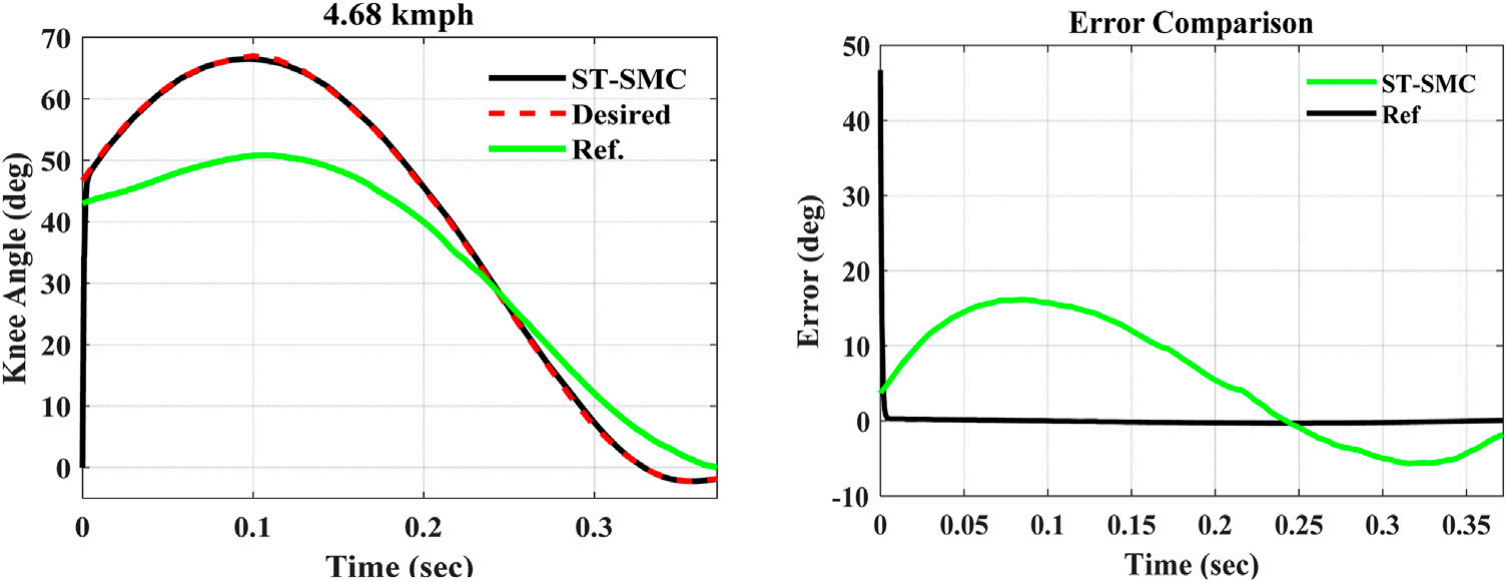
Comparison of tracking efficiency and error with the former design.

Considering the dynamic system model, the results of trailing performance and tracking error of the controller at a *4.68 kmph* walking speed are shown in Figures 5, 6.

The simulations were performed over the swing-phase period of the gait cycle, that is, for *0.372* s. Now comparing with the simulation study result obtained from the previously designed model by Seid et al. (2016) presented in green, the results in Figure 6 are obtained. As is shown from the error comparison graph, the previous mechanically designed knee exhibited a large deviation from the desired trajectory. On the other hand, the ST-SMC-controlled MR damper with the modified dynamic model has tracked the knee angle trajectory of a healthy human being with a minimum error (between 0.2145 and –0.2981°), as seen from the error comparison graph earlier.

Now the controller’s simulation outputs of the maximum flexion angle, the period of the swing phase, the shank velocity at the end of the swing phase, and the normalized mean square error (NRMSE) can be contrasted with the former design, as seen in Table 2.

**TABLE 2:** Comparison of results with Ref Seid et al., 2016.

Simulation	Maximum knee flexion angle (deg)	Duration of the swing phase (sec)	Shank velocity (rad/sec)	NRMSE (%)
Normal	67	0.372	3.887	-
ST-SMC	66.455	0.372	0.183	1.32
Ref (Seid et al., 2016)	50.631	0.368	3.766	3.79

As is clearly shown in the table, Table 2, the ST-SMC-based prosthetic knee has shown a minimized NRMSE compared to the formerly designed one. The shank velocity by the end of the swing phase was reduced as well. This minimum angular velocity is crucial since it has to be kept as small as possible so that it will be easily stopped when it reaches the end of the swing phase without the presence of excessive impact due to the use of a rubber bumper. The knee flexion angle is also close to the desired one with a minimal error.

Instabilities occur, mainly, when amputees walk on a rough terrain, and the road irregularities will expose them to vibration, causing the controller to face instabilities. Unless the controller is robust, this causes the controller to be less efficient in mimicking the normal gait cycle of a healthy human being.

In this research, two major causes of disturbances had been applied to the system model, that is, on the vertical and horizontal hip accelerations of the amputee. When amputees face a sudden increase or decrease in their forward motion, the hip horizontal acceleration will vary considerably. Furthermore, similar variable vertical acceleration will occur as amputees face irregularities on their walkways. For this evaluation, the ideal hip horizontal and vertical accelerations are curve-fitted by applying the following equations obtained using a curve-fitting tool on MATLAB

$$\begin{array}{c} y_{H}\left(t\right)=d_{1}\sin\left(e_{1}t+f_{1}\right)+d_{2}\sin\left(e_{2}t+f_{2}\right)+d_{3}\sin\left(e_{3}t+f_{3}\right)+d_{4}\sin\left(e_{4}t+f_{4}\right)\\ +d_{5}\sin\left(e_{5}t+f_{5}\right), \end{array}$$ (19)

$$x_{H}\left(t\right)=g_{1}\sin\left(h_{1}t+i_{1}\right)+g_{2}\sin\left(h_{2}t+i_{2}\right)+g_{3}\sin\left(h_{3}t+i_{3}\right)+g_{4}\sin\left(h_{4}t+i_{4}\right).$$ (20)

where: $\begin{array}{l} d_{1}=4.69,\;e_{1}=18.8\;f_{1}=1.071,\\ d_{2}=1.242,\;e_{2}=58.65,\;f_{2}=-2.299,\\ d_{3}=9.161,\;e_{3}=0.1353,\;f_{3}=-3.09,\\ d_{4}=0.4311,\;e_{4}=35.69,\;f_{4}=0.5278,\\ d_{5}=1.108,\;e_{5}=62.49,\;f_{5}=0.3738. \end{array}$ $\begin{array}{l} g_{1}=23.15,\;h_{1}=40.33,\;i_{1}=-4.523,\\ g_{2}=1.839,\;h_{2}=13.26,\;i_{2}=-1.685,\\ g_{3}=22.55,\;h_{3}=41.25,\;i_{3}=4.68,\\ g_{4}=0.6321,\;h_{4}=55.04,\;i_{4}=5.004. \end{array}$

**FIGURE 7:**
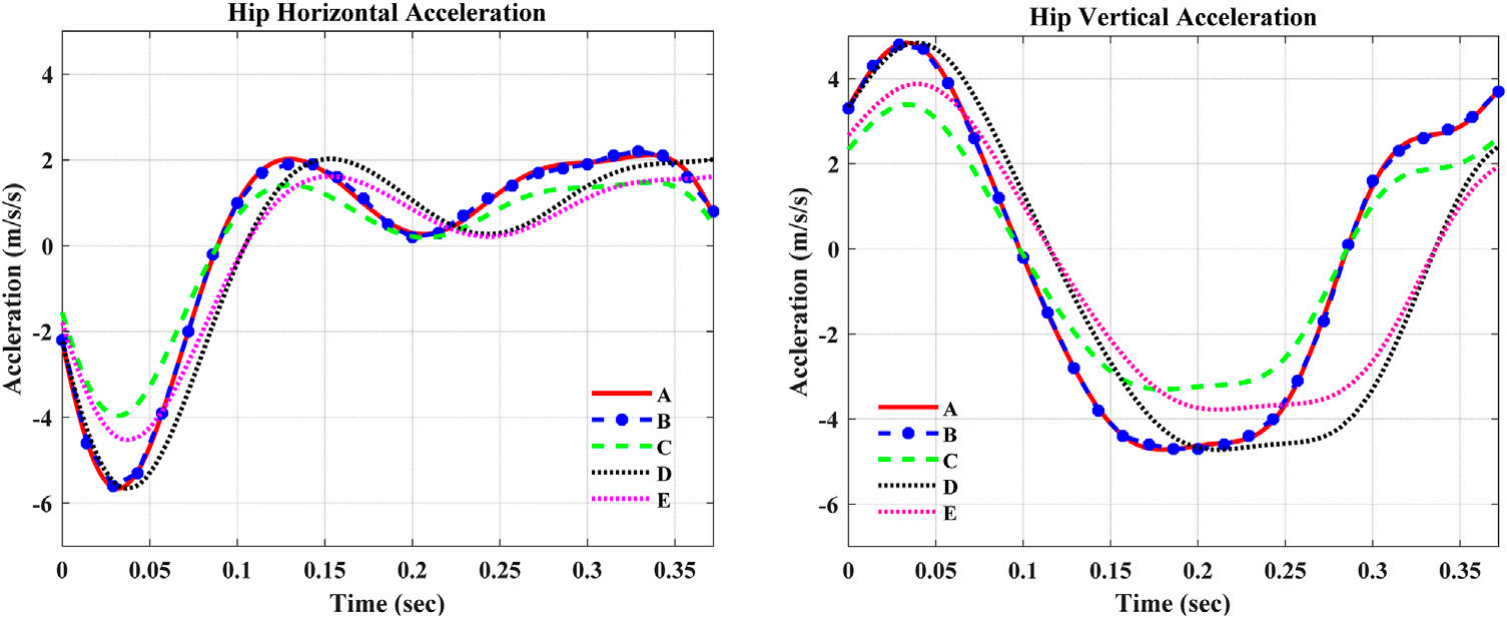
Curve-fitted and scaled data of hip acceleration. In the figures, A—Curve-fitted data (without scaling), B—Experimental data from Winter (2009), C—Curve-fitted data with 70% amplitude scaling, D—Curve-fitted data with 85% frequency scaling, and E—Curve-fitted data with 80% amplitude and 85% frequency scaling.

To represent the disturbances that occurred due to the aforementioned reasons, the amplitudes and frequencies of the aforementioned curve-fitted formulas of hip accelerations are scaled independently and simultaneously as plotted in Figure 7.

The robustness evaluation of ST-SMC was performed considering a disturbance due to the rough walking terrain as shown in Table 3. The analysis was performed by applying the amplitude and frequency scaling on the curve-fitted experimental data of vertical and horizontal hip accelerations. The analysis outputs depicted that the ST-SMC was exceptionally robust and stable, exhibiting a minimal error in tracking the desired swing-phase knee angle trajectory.

**TABLE 3:** Robustness evaluation.

Simulation	Scaling	Maximum knee flexion angle (deg)	Duration of the swing phase (sec)	Shank velocity (rad/sec)	NRMSE (%)
Normal	-	67	0.372	3.887	-
Hip horizontal acceleration					
ST-SMC	Curve-fitted	66.451	0.372	0.184	1.536
	70% amplitude scaling	66.454	0.372	0.066	1.547
	85% frequency scaling	66.455	0.372	0.426	1.556
	85% frequency and 80% amplitude scaling	66.501	0.372	0.374	1.586
Hip vertical acceleration					
ST-SMC	Curve-fitted	66.455	0.372	0.177	1.567
	70% amplitude scaling	66.453	0.372	0.139	1.503
	85% frequency scaling	66.454	0.372	0.225	1.560
	85% frequency and 80% amplitude scaling	66.500	0.372	0.176	1.613
Both vertical and horizontal hip acceleration					
ST-SMC	60% amplitude and 60% frequency scaling	65.001	0.372	0.353	1.876

**FIGURE 8:**
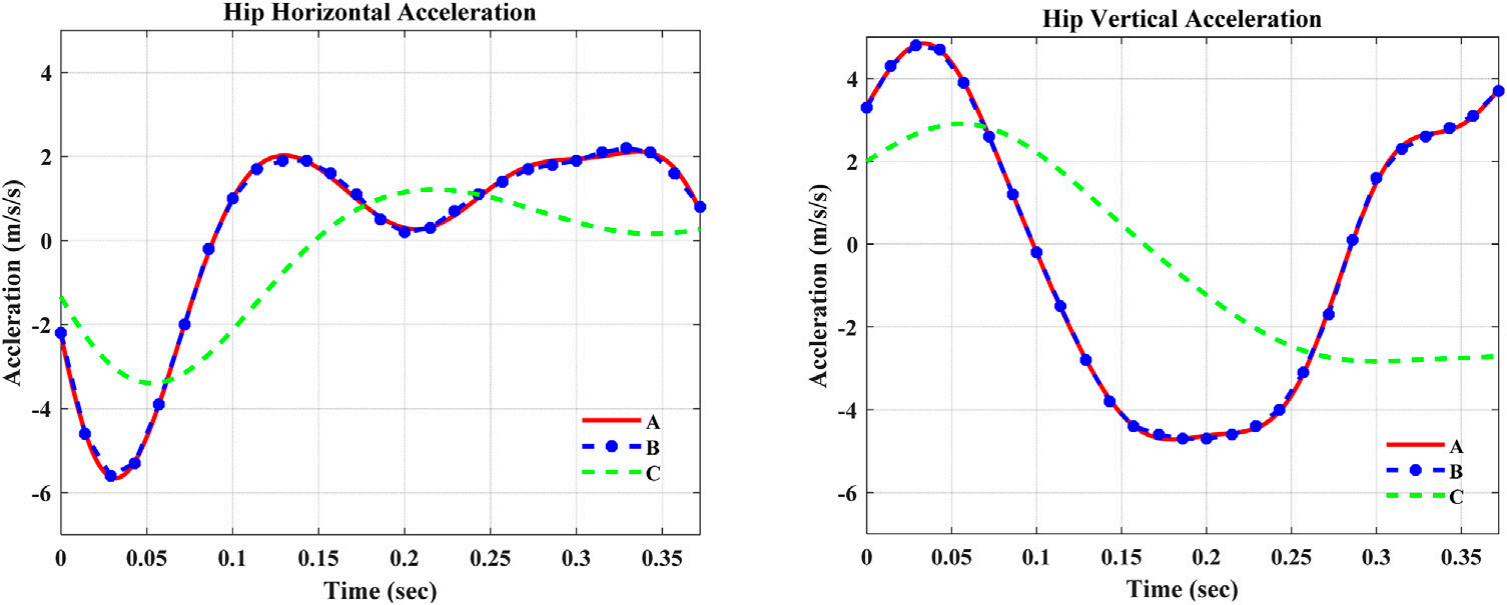
Curve-fitted and scaled data of hip acceleration. In the figures, A—Curve-fitted data (without scaling), B—Experimental data from Winter (2009), and C—Curve-fitted data with 60% amplitude and frequency scaling.

The simulation outputs in Table 3 show that the super twisting SMC was robust against the applied disturbances in the swing phase of the gait cycle. It was seen that the controller continues its tracking with a minimum deviation, that is, a small NRMSE, from the desired knee angle trajectory. On the other hand, further investigation of robustness was made by manipulating both the horizontal and vertical accelerations at the same time. The super twisting SMC still maintains its robustness with a minimum NRMSE and end phase velocity. Figure 8 shows the 60% amplitude and frequency scaling of hip accelerations.

**FIGURE 9:**
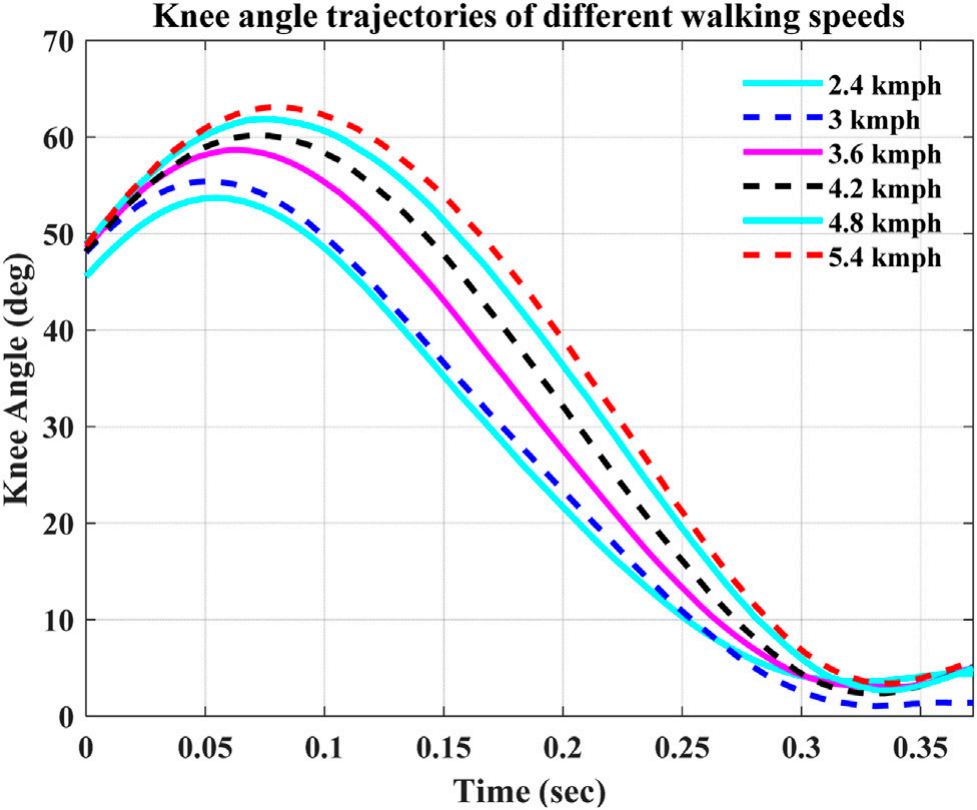
Knee angle trajectories at different walking speeds (Ekkachai et al., 2014).

Furthermore, the various walking speed performances of the ST-SMC showed that it keeps tracking the desired knee angle trajectory at those various walking speeds with a minimized tracking error. Figure 9 shows the knee angle trajectory of a healthy human gait over the course of the swing phase.

In Table 4, the NRMSE was calculated to find the error depicted by the controller while operating at different walking speeds. The results will be compared with the former design as well.

**TABLE 4:** NRMSE comparison.

Speed (kmph)	NRMSE (%)	
	ST-SMC	Ref (Ekkachai et al., 2014)
2.4	1.144363386	1.35
3	1.374520446	2.21
3.6	1.576099908	2.07
4.2	1.478942424	2.61
4.8	1.469834985	1.14
5.4	1.703841078	1.92

As is seen from the NRMSE calculation, the developed controller on the modified system model showed a better efficiency in tracking the desired knee angles at different speeds. On the other hand, the comparison of the controllers with previously designed prosthetic knees shows that the ST-SMC provided a better result while tracking the desired knee angle with a minimum error.

## 6 Discussion

The computer simulation findings of the controller applied on the modified mathematical models are presented. It is seen that at a walking speed of 4.68 kmph, the designed controller displayed an excellent performance as compared to the results found in the former mechanically designed prosthetic knees by Seid et al. (2016). The evaluation of various speed efficiencies of controllers was also executed and compared with previous work by Ekkachai et al.(2014).

Furthermore, the robustness of ST-SMC was evaluated considering a disturbance due to the rough walking terrain. The analysis was performed by applying the amplitude and frequency scaling on the curve-fitted experimental data of vertical and horizontal hip accelerations. The analysis outputs depicted that the ST-SMC was exceptionally robust compared to the previous design by Seid et al. (2016) and stable, exhibiting a minimal error in tracking the desired swing-phase knee angle trajectory of a healthy human being.

## 7 Conclusion

This study presented the design and simulation of ST-SMC for a transfemoral prosthetic knee. The dynamic system models and the MR damper model were developed using the Lagrangian mechanics and Bingham Plastic model, respectively. The relation between the yield shear stress and input current of MRF is obtained from an optimized MR damper valve design. It was observed that super twisting SMC provided a superior tracking efficiency with a reduced NRMSE of 1.32 and chattering compared to the previously designed prosthetic knee. In addition, the final shank velocity of the prosthetic knee was reduced to 0.183 rad/sec and the maximum flexion angle near to the natural gait.

Moreover, the measure of the robustness of the designed ST-SMC was performed by applying different walking speeds and several disturbances related to the rough terrain. This research had a significant contribution in designing a more stable system model controlled with ST-SMC showing negligible chattering and increased robustness. Future work includes evaluating the system with other controllers such as adaptive SMC, nonlinear PID, and integral SMC to minimize exhibited errors. Furthermore, experimental investigation and prototyping of the designed controller will be conducted.

## Data Availability

The original contributions presented in the study are included in the article/Supplementary Material, further inquiries can be directed to the corresponding author.
